# Supplementary figures and images for: TGFBR3L—An Uncharacterised Pituitary Specific Membrane Protein Detected in the Gonadotroph Cells in Non-Neoplastic and Tumour Tissue
Source: Cancers (Basel). 2020 Dec 31;13(1):114. doi: 10.3390/cancers13010114 (PMC7795056; doi:10.3390/cancers13010114)

gonadotroph

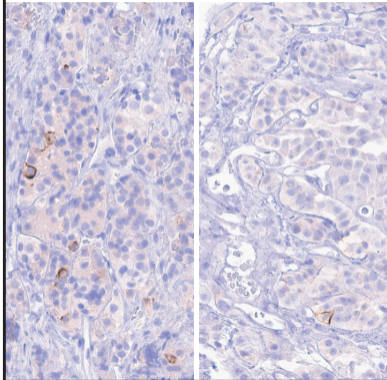

corticotroph

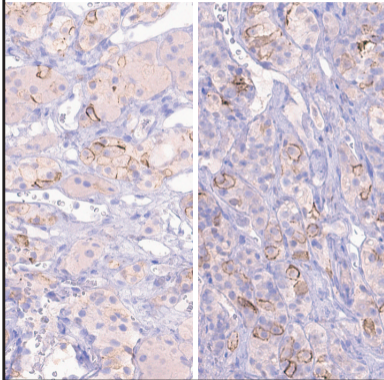

lactotroph

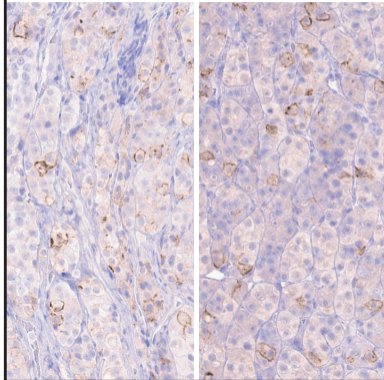

somato-lactotroph

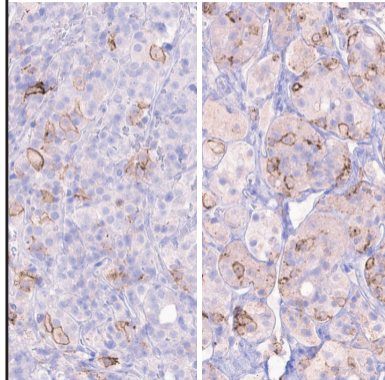

Supplement: Supplementary file 1 [file cancers-13-00114-s001.pdf]
